# ABR findings in inner ear anomaly subgroups: Influence of cochlear nerve deficiency

**DOI:** 10.1007/s00405-026-10029-x

**Published:** 2026-02-16

**Authors:** Ayşegül Eşdoğan, Eylem Saraç Kaya, Bensu Akcin, Gonca Sennaroğlu

**Affiliations:** 1 Faculty of Health Sciences, Department of Audiology, KTO Karatay University, Konya, Turkey 42020; 2 Institute of Health Sciences, Doctorate Program of Audiology, Hacettepe University, Ankara, Turkey 06100; 3 Faculty of Health Sciences, Department of Audiology, Lokman Hekim University, Ankara, Turkey 06510; 4 Faculty of Health Sciences, Department of Audiology, Hacettepe University, Ankara, Turkey 06100

**Keywords:** Inner ear anomalies, Cochlear nerve deficiency, Auditory brainstem response, Audiological findings

## Abstract

**Purpose:**

This study aimed to investigate auditory brainstem response (ABR) findings across subgroups of inner ear anomalies and to compare the ABR profiles in cases with cochlear nerve deficiency (CND).

**Methods:**

A total of 189 ears from 109 individuals with various inner ear anomalies were evaluated. Radiological findings were used to classify each ear into specific anomaly subgroups and to assess cochlear nerve status. ABR outcomes were then analyzed accordingly.

**Results:**

CND was consistently observed in complete labyrinthine aplasia, rudimentary otocyst, cochlear aplasia, and common cavity malformations, with absent ABR wave V and cochlear microphonic (CM) responses. Cochlear hypoplasia and incomplete partition subtypes showed variable ABR and CM responses. In cochlear aperture anomalies, CND was consistently observed, with absent wave V but detectable CM of variable latency. Wave V responses differed significantly between normal and CND ears (p < 0.001), whereas CM responses did not (p = 0.055).

**Conclusions:**

This study delineates ABR characteristics specific to inner ear anomaly subgroups and highlights the influence of CND on ABR outcomes. The findings underscore the potential of detailed ABR analysis to serve as a valuable tool for guiding auditory rehabilitation decisions and emphasize its relevance in shaping future clinical practice guidelines.

## Introductıon

Inner ear anomalies are observed in approximately 20% of congenital sensorineural hearing loss cases [1].These anomalies, which affect the bony labyrinth, can be diagnosed using computed tomography (CT) and magnetic resonance imaging (MRI) techniques [2]. According to the classification proposed by Sennaroglu and Bajin [3], inner ear anomalies are categorized into eight subgroups—complete labyrinthine aplasia, rudimentary otocyst, cochlear aplasia, common cavity, cochlear hypoplasia (CH) (Types I–IV), incomplete partition (IP) (Types I–III), enlarged vestibular aqueduct (EVA), and cochlear aperture anomalies—each of which presents with distinct audiological findings [4].

Auditory brainstem response (ABR) testing is an objective measure that evaluates the function of the auditory pathway from the auditory nerve to the midbrain. By enabling the assessment of synchronous neural activity, ABR plays a critical role in clinical applications and in decision-making regarding appropriate treatment strategies [5]. However, only a limited number of studies have reported ABR findings in individuals with inner ear anomalies. Moreover, the existing studies have primarily focused on the mere presence or absence of ABR responses, without providing a detailed analysis of specific ABR patterns [6–8]. To the best of our knowledge, no study in the literature has systematically investigated ABR findings across different subgroups of inner ear anomalies.

Magnetic resonance imaging (MRI) allows detailed evaluation of the presence and size of the vestibular cochlear nerve [9]. Cochlear nerve deficiency (CND), describing aplastic or hypoplastic nerves, is reported in about 18% of children with sensorineural hearing loss and may be associated with inner ear anomalies or normal canal morphology [3, 9, 10]. However, only limited audiological findings have been reported in this patient group [3, 10, 11].

The primary aim of this study was to analyze ABR findings across subgroups of inner ear anomalies, providing insight into audiological evaluation and intervention planning. The secondary aim was to assess cochlear nerve status and compare ABR results based on the presence of CND, enhancing understanding of its impact and contributing to the limited literature on this population.

## Materıal & method

This study was approved by the Local Ethical Committee (approval code: SBA 23/138) and was conducted in accordance with the Declaration of Helsinki.

### Participants

This study included individuals diagnosed with an inner ear anomaly in at least one ear based on radiological imaging, who underwent ABR testing at the Hacettepe University Audiology Clinic between January 2010 and July 2023. Inclusion criteria were the availability of temporal bone CT and MRI results, radiological confirmation of the anomaly and cochlear nerve status, and accessible ABR records from clinic archives.

Participants who met the inclusion criteria were enrolled in the study regardless of age or sex. Data were analyzed on an ear-by-ear basis, and the age of the participants at the time of ABR testing was taken into account.

### ABR evaluation

All ABR recordings were obtained using a Vivosonic Integrity™ device in a sound-attenuated room, with electrodes placed according to the 10–20 system (impedance < 5 kΩ, inter-electrode difference < 2 kΩ). Although both click and tone-burst stimuli were used, analyses focused on click-evoked responses, as these were available for all participants. Clicks (100 μs, rarefaction polarity) were delivered via ER-3A insert earphones at 27.5/s or 37.7/s, with intensities from 20 to 99 dB nHL. Cochlear microphonic (CM) responses were recorded using both rarefaction and condensation polarities. Each ipsilateral response was averaged over 2,000 sweeps, and reliability was confirmed by duplicate recordings.

All traces were independently reviewed by two audiologists. The presence of wave V was first determined; if observed, its threshold and absolute latency were recorded. In cases where wave V was absent, CM responses were analyzed—when available in both polarities—and their latencies measured if present.

### Radiological assessment

Radiological evaluations were conducted by the Department of Radiology at Hacettepe University. All participants underwent high-resolution temporal bone computed tomography (CT) and 1.5 T magnetic resonance imaging (MRI). Cochlear nerve status was assessed using axial Constructive Interference in Steady State (CISS) MRI sequences: nerves markedly smaller than the ipsilateral facial nerve were classified as hypoplastic, and those not visible were classified as aplastic. Radiological results were retrospectively reviewed, and inner ear anomalies was classified according to the system proposed by Sennaroglu and Bajin [3]. Cochlear nerve status was recorded as normal, hypoplastic, or aplastic, and ears were grouped as normal or CND.

### Statistical analysis

The data were analyzed using IBM SPSS Statistics version 25.0. Quantitative variables were expressed as mean ± standard deviation (x̄ ± SD) if normally distributed, and as median [IQR] if not normally distributed. Homogeneity of variances, as an assumption of parametric tests, was assessed using Levene’s test, while normality was evaluated with the Shapiro–Wilk test. Student’s t-test or Mann–Whitney U test was used for two-group comparisons, and categorical variables were analyzed with Chi-square test. A p-value of < 0.05 was considered statistically significant.

In this study, ChatGPT 5.0 program was used to improve the language of the article.

## Results

A total of 109 participants, including 54 females and 55 males, were enrolled in the study. Assessments were performed on an ear-by-ear basis, yielding a total of 189 ears for evaluation. The ears were classified according to the relevant inner ear anomaly groups, and the ABR findings were examined accordingly. In addition, the ears were categorized based on cochlear nerve status, and ABR findings were compared.

### ABR findings according to types of inner ear anomalies

#### Complete labyrinthine aplasia

A total of 9 ears with complete labyrinthine aplasia (7 females, 2 males; 5 right ears, 4 left ears) were examined. Cochlear nerve aplasia was observed in 8 of these ears, while cochlear nerve hypoplasia was present in 1 ear. The age of the individuals ranged from 6 to 32 months (mean: 18.55 ± 11.94 months). In this group, no Wave V or CM responses were observed at 99 dB nHL in any of the ears.

#### Rudimentary otocyst

A total of 4 ears diagnosed with rudimentary otocyst (2 females, 2 males; 3 left ears, 1 right ear) were examined. Cochlear nerve aplasia was present in all ears within this anomaly group. The age of the individuals ranged from 4 to 20 months (mean: 13.75 ± 7.76 months). In this group, no Wave V or CM responses were obtained at 99 dB nHL in any of the ears.

#### Cochlear aplasia

A total of 7 ears with cochlear aplasia (4 males, 3 females; 4 right ears, 3 left ears) were examined. Based on MRI findings, cochlear nerve aplasia was observed in 5 ears, while an unsegmented single vestibulocochlear nerve was present in 2 ears. The age of the individuals at the time of ABR testing ranged from 4 to 36 months (mean: 17.42 ± 10.43 months). In this group, no Wave V or CM responses were obtained at 99 dB nHL in any of the ears.

#### Common cavity

A total of 7 ears with common cavity anomaly (4 males, 3 females; 4 right ears, 3 left ears) were examined. MRI findings revealed an unsegmented single vestibulocochlear nerve in 4 ears, cochlear nerve hypoplasia in 1 ear, and a normal cochlear nerve in 2 ears. The age of the individuals ranged from 4 to 36 months (mean: 20.57 ± 13.15 months). In this group, no Wave V or CM responses were obtained at 99 dB nHL in any of the 7 ears.

#### Cochlear hypoplasia

Cases with cochlear hypoplasia anomaly were examined by dividing them into subgroups; CH Type I, II, III and IV.

**Fig. 1:**
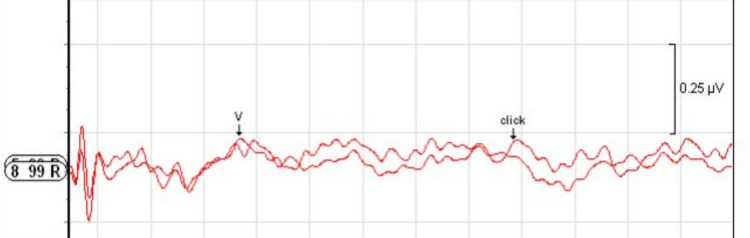
Wave V obtained at 99 dB nHL in the ear with CH Type II anomaly

**Fig. 2:**
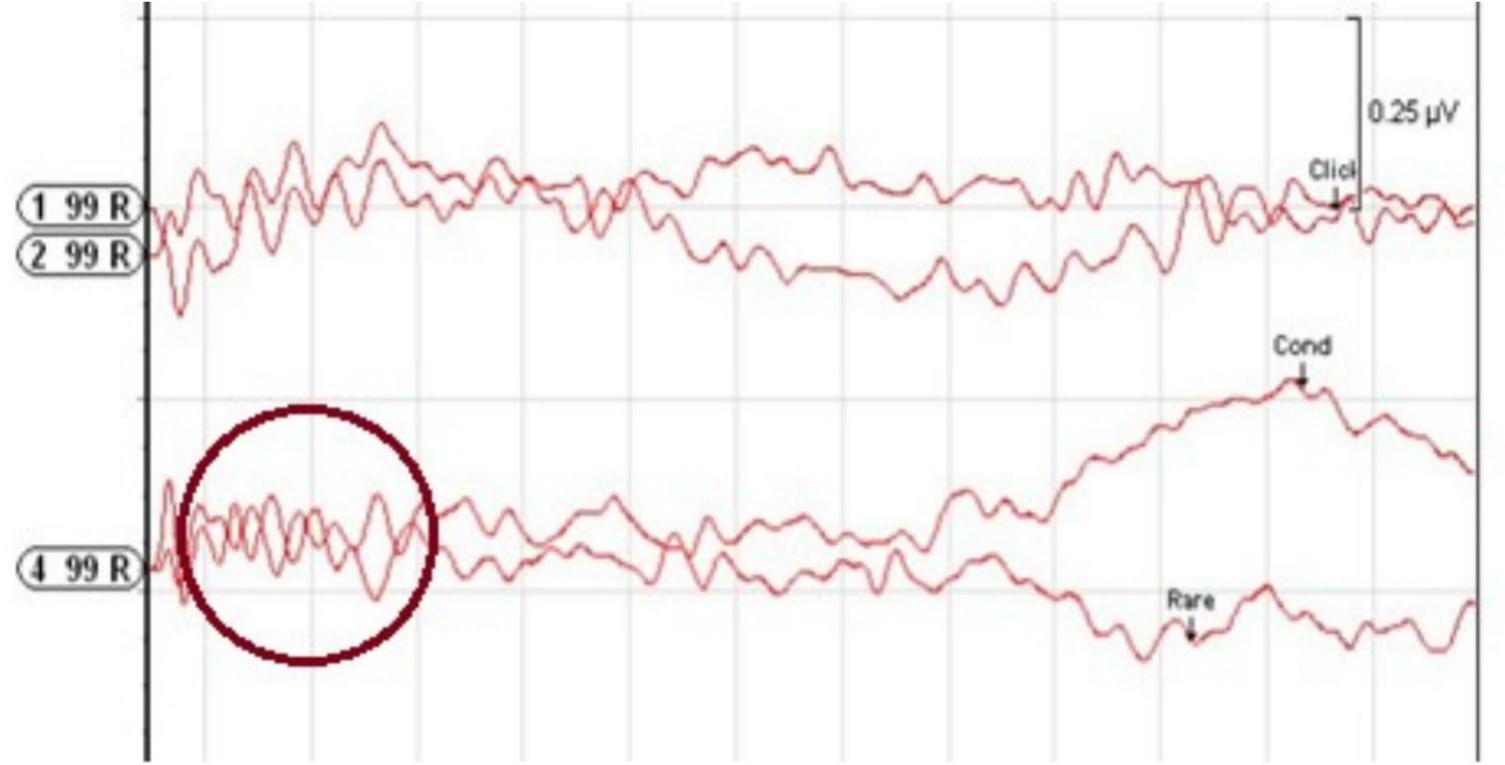
CM image obtained from the ear with CH Type III anomaly

**Fig. 3:**
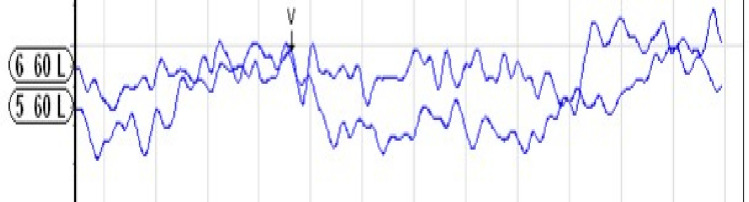
Wave V obtained at 60 dB nHL in the ear with CH Type IV anomaly

- **Cochlear Hypoplasia Type I (CH Type I):** A total of 14 ears with CH Type I anomaly (9 males, 5 females; 7 right ears, 7 left ears) were examined. According to MRI findings, 1 ear had a normal cochlear nerve, 5 ears had cochlear nerve hypoplasia, 7 ears had cochlear nerve aplasia, and 1 ear had an unsegmented single vestibulocochlear nerve. The age of the individuals in this group ranged from 1 to 64 months (mean: 19.64 ± 18.26 months). In none of the examined ears were Wave V or CM responses obtained at 99 dB nHL.
- **Cochlear Hypoplasia Type II (CH Type II):** A total of 11 ears with CH Type II anomaly (9 females, 2 males; 6 left ears, 5 right ears) were examined. Regarding cochlear nerve status, 5 ears (45.5%) showed nerve hypoplasia, 3 ears (27.3%) had nerve aplasia, and 3 ears (27.3%) had a normal cochlear nerve. The age of the individuals in this group ranged from 19 to 72 months (mean: 36.09 ± 20.51 months). In this anomaly group, Wave V at 99 dB nHL was obtained in only one ear with cochlear nerve hypoplasia, with a wave latency of 5.34 ms (Fig. 1). No Wave V or CM responses were observed at 99 dB nHL in the other ears.
- **Cochlear Hypoplasia Type III (CH Type III):** A total of 24 ears with CH Type III anomaly (15 females, 9 males; 13 right ears, 11 left ears) were examined. Regarding cochlear nerve status in this anomaly group, 5 ears had a normal cochlear nerve, 11 ears showed nerve aplasia, and 8 ears exhibited nerve hypoplasia. The age of the individuals ranged from 10 to 52 months (mean: 29.83 ± 13.29 months). In ABR evaluations, Wave V responses were obtained in 5 of the 24 ears (20.8%) with CH Type III anomaly. All 5 ears with detectable Wave V had normal cochlear nerves. The Wave V thresholds ranged from 40 to 85 dB nHL, with a mean threshold of 65 ± 18.02 dB nHL. The absolute latency of Wave V ranged from 7.40 to 9.20 ms (mean: 8.37 ± 0.81 ms). Among the remaining 17 ears in which Wave V was not obtained and CM assessment was performed, only one ear exhibited a CM response extending up to 4 ms upon polarity change at 99 dB nHL (Fig. 2).
- **Cochlear Hypoplasia Type IV (CH Type IV):** A total of 2 ears with CH Type IV anomaly (2 males; 1 right ear, 1 left ear) were examined. Normal cochlear nerves were present in both ears. The mean age at the time of ABR testing was 36 months. In one ear, a Wave V response was obtained at 60 dB nHL with a latency of 7.27 ms (Fig. 3). In the other ear, no Wave V response was obtained at 99 dB nHL, and no CM response was observed upon polarity change.

#### Incomplete partition anomalies

Cases with IP anomaly were examined by subdividing them into subgroups; IP Type I, II and III.

- **Incomplete Partition Type I (IP-I): **A total of 16 ears with IP-I anomaly (8 females, 8 males; 9 left ears, 7 right ears) were examined. Regarding cochlear nerve status, 6 ears showed nerve aplasia, 4 ears had a normal cochlear nerve, and 6 ears exhibited nerve hypoplasia. The mean age at the time of ABR testing was 33.00 ± 18.98 months (range: 4–72 months).

**Fig. 4:**
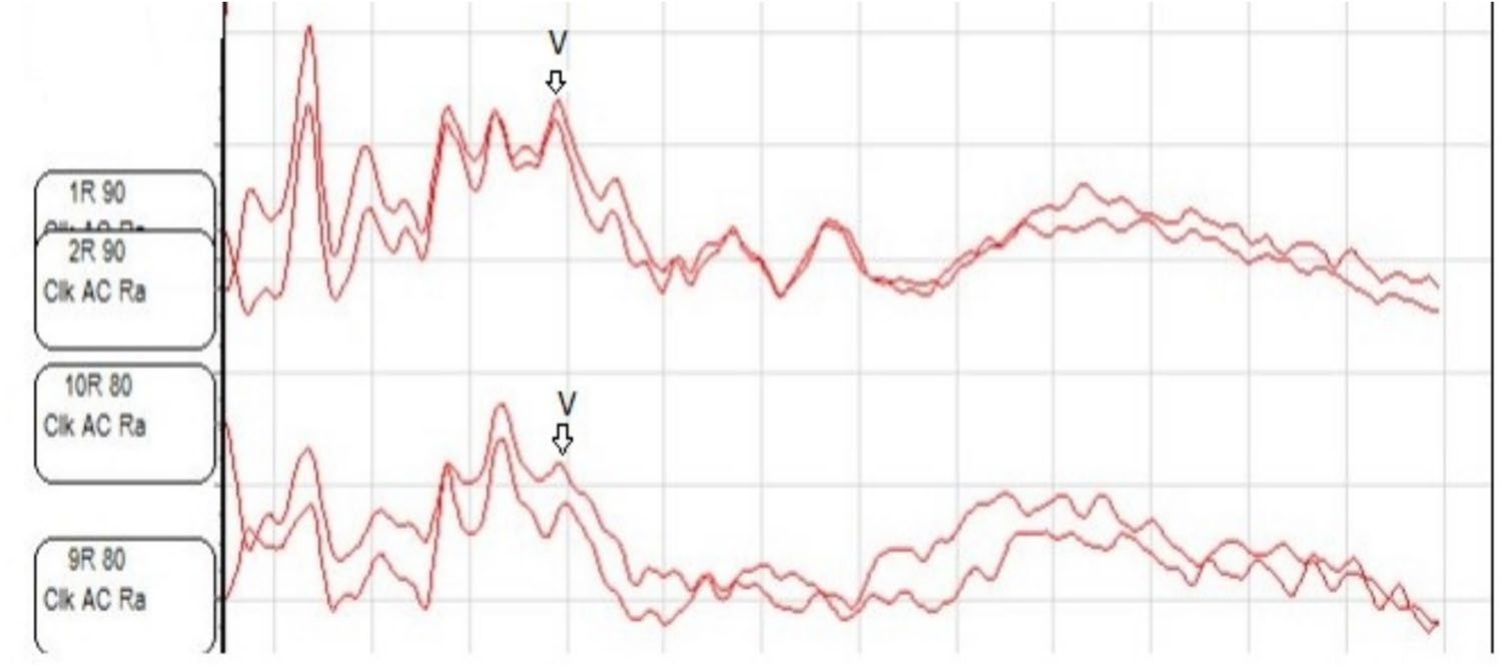
Wave V obtained at 80 dB nHL in the ear with IP-I anomaly

In ABR evaluations, Wave V response was obtained in only one ear with a normal cochlear nerve, at an 80 dB nHL threshold and 6 ms latency (Fig. 4). In the remaining ears, no Wave V responses were obtained at 99 dB nHL. Among the ears without Wave V responses, only one ear showed a CM response at 99 dB nHL with a latency of 2.5 ms.

- **Incomplete Partition Type II (IP-II):** A total of 73 ears with IP-II anomaly (43 males, 30 females; 37 right ears, 36 left ears) were examined. MRI findings revealed a normal cochlear nerve in all ears. The mean age of the individuals at the time of ABR testing was 41.12 ± 35.74 months (range: 2–180 months). ABR evaluations showed that responses were obtained in 47 of the 73 ears (64.4%). The Wave V thresholds ranged from 20 to 99 dB nHL, with a mean threshold of 70.36 ± 17.61 dB nHL. The mean absolute latency of Wave V was 7.14 ± 0.88 ms, ranging from 5.24 to 9.23 ms. The Wave V thresholds and absolute latency values obtained from individuals with IP-II anomaly are presented in Table 1.

**Table 1:** Wave V findings obtained in the IP-II anomaly group

	**Threshold level (dB nHL)**									**Absolute latency (ms)**						
	**n**	**Mean**			**Min**		**Max**		** ± SD**	**Mean**		**Min**		**Max**		** ± SD**
**Female**	21		70,66	50		99		12,36		6,90	5,24		8,94		0,84	
**Male**	26		70,11	20		99		21,17		7,34	6,13		9,23		0,88	
**Right**	21		69,42	30		99		19,58		7,16	5,24		9,23		1,05	
**Left**	26		71,11	20		99		16,19		7,13	5,79		8,73		0,73	

dB nHL: desibel normalized hearing level, ms: milisecond, n: mumber of ears, Min: minimum, Max: maximum, SD: standard deviation

Among the 47 ears with ABR responses in this anomaly group, 26 were left ears and 21 were right ears. In the left ears, the mean Wave V threshold was 71.11 ± 16.19 dB nHL, and the mean absolute latency was 7.13 ± 0.73 ms. In the right ears, the mean Wave V threshold was 69.42 ± 19.58 dB nHL, and the mean absolute latency was 7.16 ± 1.05 ms. No statistically significant differences were observed between the right and left ears in terms of mean Wave V thresholds (p = 0.748) or mean absolute Wave V latency (p = 0.916).

Regarding sex distribution, among the 47 ears with ABR responses, 21 belonged to females and 26 to males. In females, the mean Wave V threshold was 70.66 ± 12.36 dB nHL, and the mean absolute latency was 6.90 ± 0.84 ms. In males, the mean Wave V threshold was 70.11 ± 21.17 dB nHL, and the mean absolute latency was 7.34 ± 0.88 ms. No statistically signifi`cant differences were observed between sexes in terms of mean Wave V thresholds (p = 0.912) or mean absolute Wave V latency (p = 0.092).

- **Incomplete Partition Type III (IP-III):** A total of 6 ears with IP-III anomaly (3 right ears, 3 left ears) were examined, all belonging to male participants. According to MRI findings, normal cochlear nerves were present in all 6 ears. The mean age at the time of ABR testing was 22.66 ± 2.06 months (range: 20–24 months). In all 6 ears with IP-III anomaly, Wave V responses were obtained at thresholds ranging from 45 to 80 dB nHL, with a mean threshold of 62.50 ± 14.74 dB nHL. The absolute latency of Wave V ranged from 6.73 to 8.05 ms, with a mean of 7.25 ± 0.45 ms.

#### Cochlear aperture anomalies

A total of 16 ears with cochlear aperture anomaly (14 females, 2 males; 9 right ears, 7 left ears) were examined. Regarding cochlear nerve status, 7 ears showed nerve hypoplasia, and 9 ears had nerve aplasia. The mean age of the individuals in this group was 22.37 ± 13.96 months (range: 6–60 months). No ABR responses at 99 dB nHL were obtained in any of the ears in this group. CM responses were detected in 6 ears, with latencies ranging from 2 to 5 ms.

### Comparison of ABR findings according to cochlear nerve deficiency

Among a total of 189 ears diagnosed with inner ear anomalies, MRI findings revealed an unsegmented single vestibulocochlear nerve in 7 ears. The remaining 182 ears were grouped by cochlear nerve status: 86 with CND (33 hypoplastic, 53 aplastic) and 96 with a normal cochlear nerve.

Among 86 ears with CND (43 left ears, 43 right ears; 55 females, 31 males), Wave V response at 99 dB nHL and 5.34 ms was obtained in only one ear, which corresponded to a CH Type II anomaly with associated cochlear nerve hypoplasia. Among 96 ears with normal cochlear nerves (60 male, 36 female; 51 right, 45 left), Wave V responses were obtained in 60 (62.5%).A statistically significant difference was observed between the groups with normal cochlear nerves and CND in terms of Wave V response rates (p = 0.00).

Among 60 ears with ABR responses and normal cochlear nerves, 32 (53.3%) were left and 28 (46.7%) were right. Mean Wave V latency was 7.21 ± 0.75 ms in left ears and 7.27 ± 1.05 ms in right ears, with no significant difference (p = 0.790). Mean Wave V thresholds were 70.28 ± 15.69 dB nHL (left) and 67.78 ± 18.80 dB nHL (right), also without significant difference (p = 0.577).

Among 60 ears with normal cochlear nerves and ABR responses, 36 were male and 24 female. Mean Wave V threshold and latency were 68.41 ± 19.90 dB nHL and 7.37 ± 0.79 ms in males, and 70.16 ± 12.09 dB nHL and 7.05 ± 1.03 ms in females. No significant sex differences were found in thresholds (p = 0.674) or latencies (p = 0.188).

Among 73 ears with CND assessed for CM, 8 ears (10.96%) showed CM responses (mean latency 2.91 ± 1.01 ms). In 31 ears with normal nerves but no ABR, no CM responses were detected. CM response rates did not differ significantly between groups (p = 0.055).

## Discussion

The identification of structural malformations of the inner ear and the vestibulocochlear nerve plays a crucial role in the evaluation of individuals with hearing loss and in determining appropriate intervention strategies [12].Therefore, it is highly important to determine the audiological characteristics of these anomaly groups.

Only a few studies have reported ABR findings in inner ear anomalies, usually limited to response presence or absence. To the best of our knowledge, the present study is the first to comprehensively report ABR findings in inner ear anomalies by subdividing them into specific subgroups. In this study, ABR results from 189 ears of 109 individuals with inner ear anomalies were analyzed, including Wave V response rates, thresholds, latencies, and CM findings for each anomaly subgroup.

According to the database of the Department of Otolaryngology, Hacettepe University, Incomplete Partition anomalies account for 41% of inner ear anomalies [3]. In the present study group, IP-II anomaly was the most frequent (38.62%), and overall, IP anomalies constituted 50.26% of the study population.

Total labyrinthine aplasia, also known as Michel deformity, is a rare inner ear anomaly characterized by the complete absence of inner ear structures due to the arrest of otic placode development before the third gestational week [13]. In the present study, no ABR or CM responses were obtained in the total labyrinthine aplasia group. Similarly, Özgen et al. [6] reported the absence of ABR responses in this anomaly group. These findings are an expected consequence of the lack of inner ear development in total labyrinthine aplasia.

Rudimentary otocyst consists of an incomplete otic capsule of millimetric size lacking an internal acoustic canal [14]. Lu et al. [15] reported the absence of auditory brainstem responses in cases diagnosed with rudimentary otocyst. In the present study, no ABR or CM responses were obtained in any of the ears with rudimentary otocyst. The absence of ABR responses in these cases is attributed to the lack of neural connection between the otocyst and the brainstem due to the absence of internal acoustic canal development [14].

Cochlear aplasia is a type of inner ear anomaly characterized by the absence of the cochlea [3]. Jeong and Kim [16] reported that in two cases with cochlear aplasia, the auditory brainstem response did not show reproducible waveforms even at maximum stimulus intensity. In the present study, no ABR or CM responses were observed in cases with cochlear aplasia. These findings can be attributed to the underdevelopment of the cochlea.

Common cavity anomaly arises when there is a developmental arrest during the 4th gestational week, resulting in the otic capsule failing to differentiate into the primitive cochlea, vestibule, and semicircular canals [17]. In the literature, it has been reported that ABR waves cannot be obtained even at maximum stimulus intensity in this group [8, 18]. In our study, consistent with the literature, no Wave V or CM responses were obtained in the ABR testing of ears with common cavity anomaly. In contrast, Zhang et al. [17] reported that a positive ABR response was obtained in only one case with residual hearing in this group. Their study lacked detailed ABR evaluation data. While ABR responses are generally absent in common cavity anomalies, frequency-specific tone-burst stimuli may elicit responses in patients with residual hearing. In this study, only click-evoked ABR was analyzed, which may explain the absence of responses.

Cochlear hypoplasia, accounting for ~ 15% of inner ear anomalies, is defined as a smaller cochlea and classified into four subtypes [19]. Çınar et al. [20] reported varying degrees and types of hearing loss across subtypes. Similarly, our data suggest that ABR findings may differ according to cochlear hypoplasia subtypes.

Incomplete partition anomalies, characterized by a cochlea with normal external dimensions but abnormal internal structures, constitute an important subgroup of inner ear anomalies. Özbal Batuk et al. [21] in their study on 84 patients with IP, reported that the IP-I group had severe to profound hearing loss, the IP-II group had mild to profound hearing loss, and the IP-III group had severe to profound mixed hearing loss. Demir et al. [8] reported that bilateral ABR responses could not be obtained in 2 of 4 patients with IP-I anomaly using click and tonal stimuli, while ABR responses in the 80–100 dB nHL range could be obtained in only 3 of 10 patients with IP-II anomaly using click and 4000 Hz tonal stimuli. Adibelli et al. [7] reported Wave V threshold levels of 90–100 dB nHL for IP-I and IP-II anomalies, while all patients with IP-III anomaly had thresholds of 100 dB nHL. In the present study, only 1 of 16 ears with IP-I anomalies had a Wave V response. In the IP-II group, 47 of 73 ears (64.4%) had a Wave V response, with thresholds ranging from 20 to 99 dB nHL. In the IP-III group, all 6 ears showed Wave V responses (mean threshold 62.50 ± 14.74 dB nHL). These findings suggest that ABR responses vary across IP anomaly subtypes, with IP-II showing particularly variable results. Consistent with the literature, lower ABR thresholds in the IP-II group indicate better auditory outcomes.

The cochlear aperture is a bony canal transmitting the cochlear nerve from the modiolus to the internal auditory canal. Hypoplastic or aplastic formations, termed cochlear aperture anomalies, may occur alone or with other inner ear malformations. These are often associated with cochlear nerve defects, where absent ABR responses but present otoacoustic emissions can mimic auditory neuropathy in newborn screenings [22]. In the present study, a total of 16 ears with cochlear aperture anomaly were examined, each exhibiting CND. Consistent with the literature, no ABR responses were obtained in this anomaly group, although CM responses were detected in 6 ears.

The term CND is used to describe cases in which the cochlear nerve appears hypoplastic or absent on MRI scans. CND is thought to result from partial (hypoplasia) or complete (aplasia) underdevelopment of the cochlear nerve, or from post-developmental degeneration [23]. The incidence of CND in individuals with congenital sensorineural hearing loss ranges from 2.5% to 21.2% [24]. Although the literature reports that the degree of hearing loss varies from moderate to profound, particularly in individuals with cochlear nerve hypoplasia [11, 25], it has also been reported that ABR responses are generally absent in cases with CND [9, 26].

Han et al. [27] included 25 patients with bilateral severe to profound sensorineural hearing loss and CND in their study. In their ABR assessment using click stimuli, they obtained a positive ABR response in only 8 of 25 patients (32%). Miyanohara et al. [23] reported a case of a 6-year-old girl whose cochlear nerve could not be visualized on MRI of the right ear, yet pure-tone audiometry revealed moderate sensorineural hearing loss limited to high frequencies. In this case, where otoacoustic emissions were present, no ABR response was obtained at 100 dB nHL using click stimuli in the right ear. Prabhu and Shivaswamy [12] described the audiological findings of a 30-year-old adult male with bilateral cochlear nerve hypoplasia. The left cochlear nerve was thinner than the right, with no other middle or inner ear abnormalities. Audiometry showed a pure-tone average of 21.25 dB HL (right) and 62.5 dB HL (left). Click-evoked ABR at 11.1/sec and 90 dB nHL showed no responses in either ear. These findings indicate that CND affects ABR independently of pure tone audiometry, highlighting the critical role of ABR in evaluating cochlear nerve status.

Cochlear nerve deficiency can occur in isolation or together with inner ear anomalies [10, 28, 29]. In their study, Ren et al. [24] investigated the audiological characteristics of 188 ears diagnosed with CND through temporal bone CT and MRI; they obtained ABR responses in only 9% of the ears and found CM responses in 39% of them. In our study, however, ABR response was obtained in only one of 86 ears with CND (1.16%), and CM was detected in 8 of 73 ears (10.96%) that underwent CM evaluation. While an inner ear anomaly was present in all ears with CND in the present study, in the study of Ren et al.[24] inner ear anomalies were observed in only 48% (91/188) of the ears with CND. This may explain the lower rate of obtaining ABR responses in our study compared to Ren’s study.

It has been reported that ABR is also abnormal in individuals with auditory neuropathy spectrum disorder (ANSD), as in cochlear nerve anomalies [12]. ANSD is a clinical syndrome characterized by the presence of otoacoustic emissions and/or cochlear microphonics and the absence or abnormal recording of auditory brainstem responses [10]. It is estimated that approximately 10–15% of children with sensorineural hearing loss have ANSD [29]. Since ABR responses cannot be obtained and CM can be detected in children with CND, electrophysiological findings of auditory neuropathy may be observed. The presence of these findings in CND results from non-functional cochlear nerves [11]. Levi et al. [29] examined 18 children with CND (12 with aplasia and 6 with hypoplasia) and found that 13 of them showed ANSD findings in ABR testing. Buchman et al. [10] reported that, according to MRI results, 9 (18%) of 51 children who exhibited ABR findings of auditory neuropathy had CND. In the present study, 8 ears (10.96%) with cochlear nerve deficiency demonstrated ANSD findings in ABR testing, with no ABR response but detectable CM. Liddle et al. [30] reported CND in 37 (46.3%) of 80 children with ANSD, indicating a significant correlation between CND and ANSD. They also noted that the likelihood of unilateral ANSD being due to CND (57.7%) was significantly higher compared to bilateral ANSD (25%). Since CND and ANSD may present with similar findings in ABR evaluation, radiological imaging is crucial in differential diagnosis, and definitive diagnosis is established based on MRI results [10].

In this study, ABR findings were analyzed according to inner ear anomaly groups, and the impact of CND on ABR findings was demonstrated. A relatively large sample size is a strength compared to previous studies. The findings suggest that detailed ABR analysis and assessment of cochlear nerve status can guide decisions regarding auditory rehabilitation (cochlear or auditory brainstem implants) and should be considered in developing updated clinical guidelines.

## Limitations and recommendations

In this study, ABR findings for isolated EVA could not be assessed due to the lack of eligible cases. Similarly, cases with an unsegmented single vestibulocochlear nerve were excluded, as this condition was outside the study scope and case numbers were insufficient; consequently, ABR outcomes for this subgroup were not reported. Additionally, only click ABR data were available for all participants, limiting the analysis to click responses. These factors represent limitations of the present study. Future research with adequate sample sizes should include the EVA anomaly group, unsegmented vestibulocochlear nerve cases, and assessments using frequency-specific stimuli, such as tone bursts, to allow for a more comprehensive characterization of auditory brainstem responses in individuals with inner ear anomalies.

## Data Availability

Not applicable.

## References

[1] Sennaroglu L (2010) Cochlear implantation in inner ear malformations–a review article. Cochlear Implants Int 11(1):4–41. 10.1002/cii.416 19358145 10.1002/cii.416

[2] Sennaroglu L, Saatci I (2002) A new classification for cochleovestibular malformations. Laryngoscope 112(12):2230–2241. 10.1097/00005537-200212000-00019 12461346 10.1097/00005537-200212000-00019

[3] Sennaroğlu L, Bajin MD (2017) Classification and current management of inner ear malformations. Balkan Med J 34(5):397–411. 10.4274/balkanmedj.2017.0367 28840850 10.4274/balkanmedj.2017.0367 PMC5635626

[4] Sennaroglu G, Sennaroglu L (2013) Hearing loss in inner ear malformations. In: Kountakis SE (ed) Encyclopedia of Otolaryngology, Head and Neck Surgery. Springer, Berlin, Heidelberg, pp 1143–1150. 10.1007/978-3-642-23499-6_440

[5] Young A, Cornejo J, Spinner A (2023) Auditory brainstem response. In: *StatPearls* [Internet]. StatPearls Publishing, Treasure Island (FL). Available from: https://www.ncbi.nlm.nih.gov/books/NBK564321/33231991

[6] Ozgen B, Oguz K, Atas A, Sennaroglu L (2009) Complete labyrinthine aplasia: clinical and radiologic findings with review of the literature. AJNR Am J Neuroradiol 30(4):774–780. 10.3174/ajnr.A1426 19147720 10.3174/ajnr.A1426 PMC7051791

[7] Adibelli ZH, Isayeva L, Koç AM, Catlı T, Adibelli H, Olgun L (2017) The new classification system for inner ear malformations: the INCAV system. Acta Otolaryngol 137(3):246–252. 10.1080/00016489.2016.1247498 27826999 10.1080/00016489.2016.1247498

[8] Demir B, Cesur S, Şahin A, Binnetoğlu A, Çıprut A, Batman C (2019) Outcomes of cochlear implantation in children with inner ear malformations. Eur Arch Otorhinolaryngol 276(9):2397–2403. 10.1007/s00405-019-05475-9 31111254 10.1007/s00405-019-05475-9

[9] Vincenti V, Ormitti F, Ventura E, Guida M, Piccinini A, Pasanisi E (2014) Cochlear implantation in children with cochlear nerve deficiency. Int J Pediatr Otorhinolaryngol 78(6):912–917. 10.1016/j.ijporl.2014.03.003 24690223 10.1016/j.ijporl.2014.03.003

[10] Buchman CA, Roush PA, Teagle HF, Brown CJ, Zdanski CJ, Grose JH (2006) Auditory neuropathy characteristics in children with cochlear nerve deficiency. Ear Hear 27(4):399–408. 10.1097/01.aud.0000224100.30525.ab 16825889 10.1097/01.aud.0000224100.30525.ab

[11] Cinar BC, Tahir E, Batuk MO, Yarali M, Sennaroglu G, Sennaroglu L (2019) Cochlear nerve hypoplasia: audiological characteristics in children and adults. Audiol Neurootol 24(3):147–153. 10.1159/000500938 31307043 10.1159/000500938

[12] Prabhu P, Shivaswamy J (2017) Audiological findings from an adult with thin cochlear nerves. Intract Rare Dis Res 6(1):72–75. 10.5582/irdr.2016.01081 10.5582/irdr.2016.01081 PMC5359360 28357188

[13] Jackler RK, Luxford WM, House WF (1987) Congenital malformations of the inner ear: a classification based on embryogenesis. Laryngoscope 97(Suppl 40):2–14. 10.1002/lary.5540971301 3821363 10.1002/lary.5540971301

[14] Sennaroglu L, Yaralı M (2022) Rudimentary otocyst. In: Sennaroglu L (ed) Inner Ear Malformations: Classification, Evaluation and Treatment. Springer Nature Switzerland AG, Cham, pp 217–219. 10.1007/978-3-030-83674-0_20

[15] Lu S, Wei X, Kong Y, Yang B, Dhanasingh A, Li Y (2023) Cochlear implantation for rudimentary otocysts: two case reports. Otol Neurotol 44(5):e295–e299. 10.1097/MAO.0000000000003875 37167446 10.1097/MAO.0000000000003875

[16] Jeong SW, Kim LS (2012) Cochlear implantation in children with cochlear aplasia. Acta Otolaryngol 132(9):910–915. 10.3109/00016489.2012.675627 22690949 10.3109/00016489.2012.675627

[17] Zhang L, Qiu J, Qin F, Zhong M, Shah G (2017) Cochlear implantation outcomes in children with common cavity deformity: a retrospective study. J Otol 12(3):138–142. 10.1016/j.joto.2017.03.004 29937849 10.1016/j.joto.2017.03.004 PMC5963467

[18] Xia J, Wang W, Zhang D (2015) Cochlear implantation in 21 patients with common cavity malformation. Acta Otolaryngol 135(5):459–465. 10.3109/00016489.2014.990054 25677857 10.3109/00016489.2014.990054

[19] Sennaroglu L, Bajin MD, Pamuk E, Tahir E (2016) Cochlear hypoplasia type four with anteriorly displaced facial nerve canal. Otol Neurotol 37(10):e407–e409. 10.1097/MAO.0000000000001220 27662465 10.1097/MAO.0000000000001220

[20] Cinar BC, Batuk MO, Tahir E, Sennaroglu G, Sennaroglu L (2017) Audiologic and radiologic findings in cochlear hypoplasia. Auris Nasus Larynx 44(6):655–663. 10.1016/j.anl.2016.12.002 28087093 10.1016/j.anl.2016.12.002

[21] Batuk MÖ, Çınar BÇ, Özgen B, Sennaroğlu G, Sennaroğlu L (2017) Audiological and radiological characteristics in incomplete partition malformations. J Int Adv Otol 13(2):233–238. 10.5152/iao.2017.3030 28816695 10.5152/iao.2017.3030

[22] Tahir E, Özgen B, Sennaroğlu L (2022) Cochlear aperture abnormalities. In: Sennaroğlu L, editor. *Inner Ear Malformations: Classification, Evaluation and Treatment*. Springer; p. 313–324. 10.1007/978-3-030-83674-0_28

[23] Miyanohara I, Miyashita K, Takumi K, Nakajo M, Kurono Y (2011) A case of cochlear nerve deficiency without profound sensorineural hearing loss. Otol Neurotol 32(4):529–532. 10.1097/MAO.0b013e318210b88c 21358452 10.1097/MAO.0b013e318210b88c

[24] Ren C, Lin Y, Xu Z, Fan X, Zhang X, Zha D (2022) Audiological characteristics and cochlear implant outcome in children with cochlear nerve deficiency. Front Neurol 13:1080381. 10.3389/fneur.2022.1080381 36619922 10.3389/fneur.2022.1080381 PMC9813738

[25] Taiji H, Morimoto N, Matsunaga T (2012) Unilateral cochlear nerve hypoplasia in children with mild to moderate hearing loss. Acta Otolaryngol 132(11):1160–1167. 10.3109/00016489.2012.696781 22830941 10.3109/00016489.2012.696781

[26] Zhang Z, Li Y, Hu L, Wang Z, Huang Q, Wu H (2012) Cochlear implantation in children with cochlear nerve deficiency: a report of nine cases. Int J Pediatr Otorhinolaryngol 76(8):1188–1195. 10.1016/j.ijporl.2012.05.003 22664315 10.1016/j.ijporl.2012.05.003

[27] Han JJ, Suh MW, Park MK, Koo JW, Lee JH, Oh SH (2019) A predictive model for cochlear implant outcome in children with cochlear nerve deficiency. Sci Rep 9(1):1154. 10.1038/s41598-018-37014-7 30718613 10.1038/s41598-018-37014-7 PMC6362156

[28] Adunka OF, Jewells V, Buchman CA (2007) Value of computed tomography in the evaluation of children with cochlear nerve deficiency. Otol Neurotol 28(5):597–604. 10.1097/01.mao.0000281804.36574.72 17667769 10.1097/01.mao.0000281804.36574.72

[29] Levi J, Ames J, Bacik K, Drake C, Morlet T, O’Reilly RC (2013) Clinical characteristics of children with cochlear nerve dysplasias. Laryngoscope 123(3):752–756. 10.1002/lary.23636 23086614 10.1002/lary.23636

[30] Liddle K, Fitzgibbons EJ, Beswick R, Driscoll C (2022) Cochlear nerve deficiency is an important cause of auditory neuropathy spectrum disorder at a population level in children. Int J Pediatr Otorhinolaryngol 158:111171. 10.1016/j.ijporl.2022.111171 35552163 10.1016/j.ijporl.2022.111171

